# Emotional Intelligence, Motivational Climate and Levels of Anxiety in Athletes from Different Categories of Sports: Analysis through Structural Equations

**DOI:** 10.3390/ijerph15050894

**Published:** 2018-05-01

**Authors:** Manuel Castro-Sánchez, Félix Zurita-Ortega, Ramón Chacón-Cuberos, Carlos Javier López-Gutiérrez, Edson Zafra-Santos

**Affiliations:** 1 Department of Didactics of Musical, Plastic and Corporal Expression, University of Almería, 04120 Almería, Spain; 2 Department of Didactics of Musical, Plastic and Corporal Expression, University of Granada, 18071 Granada, Spain; felixzo@ugr.es (F.Z.-O.); cjlopez@ugr.es (C.J.L.-G.); 3 Department of Integrated Didactics, University of Huelva, 21007 Huelva, Spain; ramon.chacon@ddi.uhu.es; 4 Kinesiology School, University Santo Tomas, 837003 Santiago de Chile, Chile; ezafra@santotomas.cl

**Keywords:** emotional intelligence, motivational climate, anxiety, sport

## Abstract

(1) Background: Psychological factors can strongly affect the athletes’ performance. Therefore, currently the role of the sports psychologist is particularly relevant, being in charge of training the athlete’s psychological factors. This study aims at analysing the connections between motivational climate in sport, anxiety and emotional intelligence depending on the type of sport practised (individual/team) by means of a multigroup structural equations analysis. (2) 372 semi-professional Spanish athletes took part in this investigation, analysing motivational climate (PMCSQ-2), emotional intelligence (SSRI) and levels of anxiety (STAI). A model of multigroup structural equations was carried out which fitted accordingly (χ^2^ = 586.77; df = 6.37; *p* < 0.001; Comparative Fit Index (CFI) = 0.951; Normed Fit Index (NFI) = 0.938; Incremental Fit Index (IFI) = 0.947; Root Mean Square Error of Approximation (RMSEA) = 0.069). (3) Results: A negative and direct connection has been found between ego oriented climate and task oriented climate, which is stronger and more differentiated in team sports. The most influential indicator in ego oriented climate is intra-group rivalry, exerting greater influence in individual sports. For task-oriented climate the strongest indicator is having an important role in individual sports, while in team sports it is cooperative learning. Emotional intelligence dimensions correlate more strongly in team sports than in individual sports. In addition, there was a negative and indirect relation between task oriented climate and trait-anxiety in both categories of sports. (4) Conclusions: This study shows how the task-oriented motivational climate or certain levels of emotional intelligence can act preventively in the face of anxiety states in athletes. Therefore, the development of these psychological factors could prevent anxiety states and improve performance in athletes.

## 1. Introduction

Currently, sports psychology focuses on the analysis of diverse psychological variables and cognitive processes affecting the athletes’ performance, concentrating on improving their cognitive abilities with the intention of maximising efficiency. In this sense, it makes it necessary to study certain variables such as motivational climate, leadership, anxiety, emotional intelligence or resilience due to their great influence on athletes’ performance, making these factors a fundamental object of study for specialists in sports psychology [1,2,3].

Motivation is one of the most studied factors in psychology, due to its great potential to explain human behaviour, as motivation influences certain actions, modifying their intensity and direction [4]. Research on motivation in this study is based on the Self-determination Theory [5] and the Achievement Goal Theory [6], focusing on motivational climate, which represents the set of indicators perceived by individuals in their sports environment, by means of which failure or success will be defined [7]. This theory focuses on the idea that coaches can create motivational climates oriented towards task (mastery) or ego (performance) depending on the success criteria chosen [8]. If the coach concentrates on result, he will be promoting a motivational climate oriented towards ego (the coach uses unequal recognition of athletes based on abilities and individual skills, therefore encouraging intragroup rivalry), whereas a task oriented climate will be promoted if the motivational climate is focused on process (the coach encourages effort and personal self-improvement, feeling of equality between members of the same team and cooperative learning) [9].

When the athlete is oriented towards task, the levels of enjoyment and pleasure performing the activity increase, focusing on the achievement of intrinsic goals. Therefore, athletes will be oriented either towards task or ego, depending to a large extent on the motivational climate promoted by the coach [10]. Nevertheless, ego oriented goals are associated with higher levels of anxiety, as athletes act in order to achieve extrinsic goals, not dependent on themselves, therefore pressure increases so as to show abilities and outperform others, thus leading to problems in their personal development [11].

Another significant psychological factor in the context of sports is anxiety, analysed by Weinberg & Gould [12] in the context of sports, and understood as a psycho-emotional negative state of mind characterised by the manifestation of worry and nervousness, connected directly with arousal and finding a cognitive and a somatic component. Arousal is an optimal state of performance which is characterised by calmness, relaxation and skill, leading to the athlete feeling that he has the ability to completely control the situation [12,13]. In this state of alert, the athlete feels optimal self-control and self-confidence, highly related to the concept of flow, isolating himself from everything and just concentrating deeply on the activity, being able to focus all his abilities, skills, thoughts and emotions on task performance [14]. Research in the context of sports has been carried out analysing control of anxiety in competitive and training contexts, discovering athletes with lower levels of anxiety get better results in competitions while individuals with high levels of anxiety obtain worse results [15].

The third psychological factor analysed in this study is emotional intelligence, defined by Salovey & Grenwal [16] as a human ability enabling the integration of cognitive and emotional aspects effectively. Perception, management and use of emotions in the context of sports strongly influence performance, but the study of emotional factors related to sport is still scarce [17]. According to recent research in this field, it is necessary to work on emotional aspects as much as on the other cognitive, physical, technical and tactical factors [18]. The athlete’s emotional state exerts a great deal of influence on the performance and development of the activity [19,20,21]. McConville et al. [22] add to this that cognitive strategies of confrontation will also strongly affect competition performance and will decrease anxiety states. The study of these psychological factors is key to understanding cognitive processes in the context of sports, so this study complements diverse others carried out in the sports environment surrounding motivational climate, anxiety and emotional intelligence in athletes from different categories [23,24,25].

Thus, literature shows the need to work and train motivational and emotional factors aiming at reducing anxiety levels and improving sport performance in athletes. In this sense, it is important to analyse the differences between these factors depending on the type of sport done—individual or collective—due to their different characteristics such as competitive density, ways of coping with adversity or influence of partners. Therefore, this research aims: (1) to define and contrast an explanatory model about motivational climate in sport, anxiety and emotional intelligence in athletes; (2) to analyse relationships between motivational climate in sport, anxiety and emotional intelligence depending on the type of sports practised (individual/collective) by means of a structural multigroup equations analysis.

## 2. Materials and Methods

### 2.1. Subjects and Design

This descriptive and cross sectional study has been carried out on a sample of 372 semi-professional Spanish athletes of both sexes (63.2% men and 36.8% women), aged between 18 and 50 (M = 21.24; SD = 3.09) who practised team sports (141 football players and 41 paddle players) and individual sports (172 runners and 18 taekwondists). The sampling was done by convenience, according to categories of sports. The sample was obtained from diverse sport clubs, eight of them running clubs, 12 football, two paddle tennis and two taekwondo clubs, requesting voluntary participation. It must be pointed out that not repeating individuals has been guaranteed in order to avoid data duplication by means of an individualised monitoring during data collection. Furthermore, it can be established that the sample size is adequate for the model developed since the method of maximum likelihood in the analysis of covariances is used. In addition, a valid coefficient is obtained for the root Mean Square Error of Approximation and the standard error bias for parameters does not exceed 5% [26].

### 2.2. Measures

Type of sport was gathered through an ad-hoc questionnaire, classifying categories of sports into: individual and collective.

Motivational Climate questionnaire (PMCSQ-2) extracted from the original version by Newton et al. [27] and adapted to Spanish by González-Cutre et al. [27]. This instrument is made up of 33 items rated using a Likert scale with 5 options ranging from 1 = strongly disagree to 5 = strongly agree. The questionnaire establishes two categories: Task Oriented Climate, with its categories, Cooperative Learning, Effort/Improvement and Important role; and Ego Oriented Climate with its corresponding categories, Punishment for Mistakes, Unequal Recognition and Rivalry between members of the Group. The internal consistency (Cronbach’s Alpha) of the instrument obtained by González-Cutre et al. [28] in its Spanish version got a α = 0.90 in Ego Oriented Climate (α = 0.77 in Punishment for Mistakes, α = 0.87 in Unequal Recognition and α = 0.61 in Rivalry between Members) and a α = 0.84 in Task oriented climate (α = 0.65 in Cooperative Learning, α = 0.70 in effort/improvement and α = 0.70 in important role). In this research study we obtained a α = 0.89 in Ego Oriented Climate (α = 0.93 in Punishment for Mistakes, α = 0.91 in Unequal Recognition and α = 0.68 in Rivalry between Members) and a α = 0.93 in Task Oriented Climate (α = 0.83 in Cooperative Learning, α = 0.84 in Effort/Improvement and α = 0.86 in Important Role).

Schutte Self Report Inventory (SSRI) is gathered from the original questionnaire by Shutte et al. [29] which measures emotional intelligence in a unifactorial solution. Adapted by García-Coll et al. [30] to the four-factors model where other dimensions are calculated such as Emotion Perception, Self-emotional Management, Heteroemotional Management (managing others’ emotions) and Emotion Utilisation. This test is a 33-item measuring instrument which evaluates the individuals’ ability to identify, understand and manage self-emotions and others’ emotions. Items are measured using a 5 option Likert scale, where 1 = strongly disagree and 5 = strongly agree. For this research study the model proposed by García-Coll et al. [30] was used, where items 5, 28 and 33 are removed. This is because the instrument was formulated in a negative way that created a kind of response factor which disrupted the whole analysis, so the instrument used in this analysis is made up of 30 items. García-Coll et al. [29] study ascertained a reliability of α = 0.91, in General Emotional Intelligence, whereas other dimensions obtained: Emotion Perception (α = 0.70), Self-emotional Management (α = 0.77), Heteroemotional Management (α = 0.78) and Emotion Utilisation (α = 0.63). In this research, it was obtained similar values such as α = 0.91 in General Emotional Intelligence, Emotion Perception (α = 0.77), Self-emotional Management (α = 0.74), Heteroemotional Management (α = 0.78) and Emotion Utilisation (α = 0.69).

Anxiety (STAI), State-Trait Anxiety Inventory has been developed based on Spielberger et al. [31] original “State-Trait Anxiety Inventory” (STAI), used for the measurement of levels of state-anxiety and trait-anxiety. This instrument is one of the most widely used in the world, commonly used in the context of health [32] and the context of sports [33]. This instrument evaluates using a Likert scale with 4 options, ranging from 0 (nothing) to 3 (very much). The questionnaire is made up of 40 items indicating two levels, State-Anxiety (anxiety produced in a certain moment and caused by a stimulus sensed as threatening) and Trait-Anxiety (long-term anxiety characterised by the persons’ tendency towards reacting regularly in an anxious way when faced with stimuli usually considered as non-stressful). Items 1 to 20 measure state-anxiety, establishing the sum of them. Questions 1, 2, 5, 8, 10, 11, 15, 16, 19 and 20 are written in the negative being necessary to reverse the scoring before analysing it. On the other hand, items 21 to 40 measure trait-anxiety, establishing the sum of them. Questions 21, 26, 27, 30, 33, 36 and 39 are written in the negative, so it is necessary to reverse the scoring. The overall rating of trait-anxiety and state-anxiety ranges between 0 and 60 points. A classification of both types of anxiety is done following the quantiles in the source version [30]. In this investigation, State-Anxiety a reliability of α = 0.92 and for Trait-Anxiety α = 0.89.

### 2.3. Procedure

The first step was to contact each of the federations of the sports analysed in this research study in order to gauge number of athletes and federated clubs. Next, researchers contacted the different clubs and athletes to explain the nature and aim of this research, thereby setting the dates when the data collection was going to take place. As soon as the clubs and athletes had accepted taking part in the study, the questionnaires were completed from September to December 2016, before the training sessions, with the intention of reaching an optimal completion. Fifty-six questionnaires were rejected as they were not correctly completed after the data collection. This research has followed the line put forward by the Declaration of Helsinki relating to research projects, as well as national legislation for clinical trials (Royal Decree 223/2004 of 6 February), law on biomedical research (Law 14/2007, of 3 July) and on the protection of personal data (Law 15/1999 of 13 December).

### 2.4. Statistical Analysis

Statistical software IBM SPSS^®^ (IBM Corp, Armonk, NY, USA) was used in its version 22.0 for Windows in order to analyse basic descriptive. Programme IBM AMOS^®^ 23 (IBM Corp, Armonk, NY, USA) was used to analyse the existing relationship between implied constructs in the structural model. Once the theoretical model is developed, an analysis of routes is carried out, considering connections of the matrix using multigroup analysis classifying participants according to type of sport as a grouping variable. Thus, two different structural models are formed, with the purpose of verifying if the connections between the studied variables vary depending on the type of sport practised: individually or collectively.

**Figure 1:**
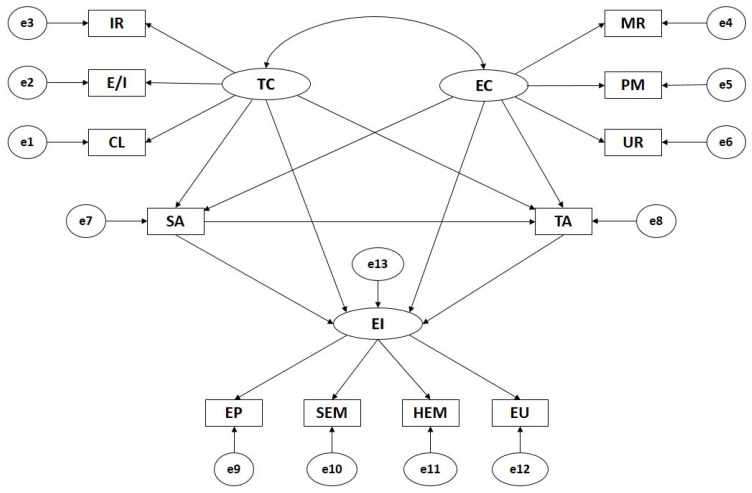
Theoretical Model. Note: TC, Task Climate; CL, Cooperative Learning; E/I, Effort/Improvement; IR, Important Role; EC, Ego Climate; MR, Member Rivalry; PM, Punishment for Mistakes; UR, Unequal Recognition; SA, State Anxiety; TA, Trait Anxiety; EI, Emotional Intelligence; HEM, Heteroemotional Management; SEM, Self-emotional management; EP, Emotion Perception; EU, Emotion Utilisation.

The path methods are made up of 12 observational variables and three latent variables in order to establish indicators (Figure 1). Causal explanations for latent variables are given based on observed relations between indicators, considering reliability of measurements. Additionally, measurement errors are included in the observable variables in order to directly control them. One-way arrows are influence lines between latent and observable variables, being interpreted as multivariate regression coefficients. Two-way arrows show connections between latent variables, also representing regression coefficients.

Task oriented motivational Climate (TC) and Ego oriented motivational Climate (EC) act as exogenous variables and each of them is inferred by three indicators. In Task Climate, the indicators are IR (Important Role), E/I (Effort/Improvement) and CL (Cooperative Learning). In Ego Climate, the indicators are PM (Punishment for Mistakes), UR (Unequal Recognition) and MR (Member Rivalry). State-anxiety (SA) and Trait-Anxiety (TA) act as endogenous variables, receiving the effects of task climate (TC) and ego climate (EC). Likewise, general emotional intelligence (EI) acts as endogenous variable, receiving the effects of State-Anxiety (SA), Trait-Anxiety (TA), Task Climate (TC) and Ego Climate (EC).

Model fit was checked with the purpose of verifying its compatibility and empirical information. Fit reliability was done according to Marsh’s goodness of fit [34]. In the case of Chi-square, non-significant values associated to *p* indicate a good model fit. The value of the Comparative Fit Index (CFI) will be acceptable with values higher than 0.90 and excellent when higher than 0.95. Normed Fit Index (NFI) should be higher than 0.90. Incremental Fit Index (IFI) will be acceptable with values higher than 0.90 and excellent when higher than 0.95. Finally, the value of the Root Mean Square Error of Approximation (RMSEA) will be excellent if lower than 0.05 and acceptable when lower than 0.08.

## 3. Results

The structural equation modelling proposed reveals a good fit in all the indices. Chi-square shows a significant value of *p* (χ^2^ = 586.77; df = 6.37; *p* < 0.001). Nevertheless, this index cannot be interpreted in a standard way, besides the problem of its sensitivity to sample size [34]. This way, other standardised fit indices are used, these being less sensitive to sample size. Comparative Fit Index (CFI) scored 0.95, being excellent. Normed Fit Index (NFI) obtained 0.94 and Incremental Fit Index (IFI) a value of 0.94, both acceptable. Root Mean Square Error of Approximation (RMSEA) obtains an acceptable value of 0.07.

**Figure 2:**
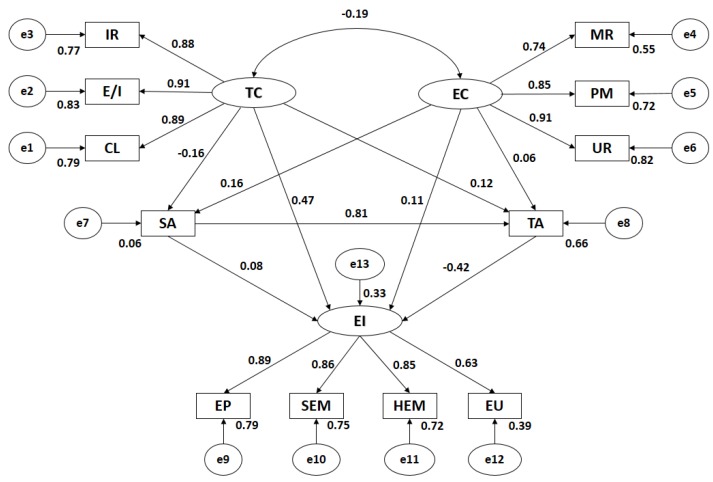
Structural equation modelling in individual sports. Note: TC, Task Climate; CL, Cooperative Learning ; E/I, Effort/Improvement; IR, Important Role; EC, Ego Climate; MR, Member Rivalry; PM, Punishment for Mistakes; UR, Unequal Recognition; SA, State Anxiety; TA, Trait Anxiety; EI, Emotional Intelligence; HEM, Heteroemotional Management; SEM, Self-emotional management; EP, Emotion Perception; EU, Emotion Utilisation.

Figure 2 and Table 1 show the estimated values for parameters in the structural model concerning athletes who practise individual sports. These must have a suitable magnitude and the effects must be significantly distinct from zero. Additionally, improper estimations such as negative variance should not be found.

By analysing influence of indicators in each latent variable in Motivational Climate, it was observed that all had statistically significant differences in level *p* < 0.005, being all relations positive and direct. In the case of Task Climate, Effort/Improvement is the indicator showing a higher correlation coefficient (*r* = 0.91), followed by Cooperative Learning (*r* = 0.89) and Important Role (*r* = 0.88). In Ego Climate, the greater association is for Unequal Recognition (*r* = 0.91), followed by Punishment for Mistakes (*r* = 0.85) and Member Rivalry (*r* = 0.74). Statistically significant relations are observed in level *p* < 0.005 between all motivational climate categories and their dimensions, which are positive and direct. The relationship between Task Climate and Ego Climate is significant at level *p* < 0.005, being negative and indirect (*r* = −0.19).

Likewise, the relationship between Motivational Climate and Anxiety is shown. Significant associations (*p* < 0.005) can be observed between Task Climate and Trait-anxiety—which is positive and direct (*r* = 0.12)–and between Task Climate and State-anxiety—which is negative and indirect (*r* = −0.16). There are also statistically significant differences at level *p* < 0.005 between Ego Climate and State-anxiety, revealing a direct association (*r* = 0.16). In the case of this achievement goal orientation and Trait-anxiety, statistically significant differences (*p* = 0.037) show a direct relation of little strength (*r* = 0.06). Additionally, State-anxiety and Trait-anxiety were positively related (*r* = 0.81; *p* < 0.005).

Finally, statistically significant relations can be observed at level *p* < 0.005 between General Emotional Intelligence and its indicators, being all associations positive and direct. It is observed that Emotion Perception is the indicator with the highest correlation (*r* = 0.89), followed by Self-emotional Management (*r* = 0.86), Heteroemotional Management (*r* = 0.85) and Emotion Utilisation (*r* = 0.63). Regarding the associations of Motivational Climate with Emotional Intelligence, statistically significant differences (*p* < 0.005) were found with Task Climate (*r* = 0.47)—being positively related–and with Trait-anxiety (*r* = −0.42)–revealing an indirect or negative relation. Emotional intelligence and Ego Climate revealed a positive and direct relation (*r* = 0.11; *p* < 0.01), while Emotional Intelligence and State-anxiety did not reveal statistically significant associations in athletes in individual categories.

**Figure 3:**
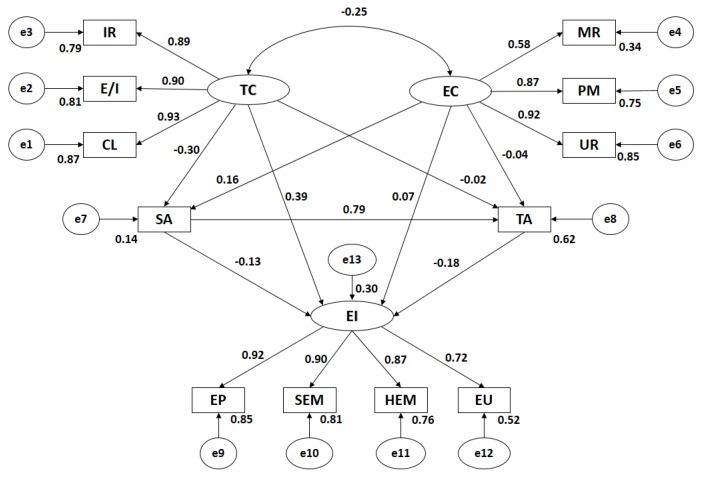
Structural equation modelling in team sports. Note: TC, Task Climate; CL, Cooperative Learning ; E/I, Effort/Improvement; IR, Important Role; EC, Ego Climate; MR, Member Rivalry; PM, Punishment for Mistakes; UR, Unequal Recognition; SA, State Anxiety; TA, Trait Anxiety; EI, Emotional Intelligence; HEM, Heteroemotional Management; SEM, Self-emotional management; EP, Emotion Perception; EU, Emotion Utilisation.

Figure 3 and Table 2 show estimate values of parameters in the structural model for athletes practising team sports. These must have a suitable magnitude and the effects must be significantly distinct from zero. Additionally, improper estimations such as negative variance should not be found.

Analysing the influence of indicators in each latent variable of Motivational Climate, it was observed that all had statistically significant differences at level *p* < 0.005, being all relations positive and direct. In the case of Task Climate, Cooperative Learning is the indicator which shows a higher correlation coefficient (*r* = 0.93), followed by Effort/Improvement (*r* = 0.90) and Important Role (*r* = 0.89). In Ego Climate, the greater association is for Unequal Recognition (*r* = 0.92), followed by Punishment for Mistakes (*r* = 0.87) and Member Rivalry (*r* = 0.58). Statistically significant relations are observed in level *p* < 0.005 between all Motivational Climate categories and their dimensions, which are positive and direct. Connection between Task Climate and Ego Climate is significant at level *p* < 0.005, being negative and indirect (*r* = −0.25).

In the same way, significant associations (*p* < 0.005) can be observed in relations between Task Climate and State-anxiety which is negative and indirect (*r* = −0.30), whereas the relation between Task Climate and Trait-anxiety is not statistically significant. Statistically significant differences are also observed at level *p* < 0.005 between Ego Climate and State-anxiety, revealing a direct association (*r* = 0.16). In the case of this goal orientation and Trait-anxiety, no statistically significant differences are obtained. Additionally, State-anxiety and Trait-anxiety were positively related (*r* = 0.79; *p* < 0.005).

Regarding Emotional Intelligence, it is observed that Emotion Perception is the indicator with the highest correlation (*r* = 0.92), followed by Self-emotional Management (*r* = 0.90), Heteroemotional Management (*r* = 0.87) and Emotion Utilisation (*r* = 0.72). Furthermore, statistically significant relations can be observed in level *p* < 0.005 between General Emotional Intelligence and its indicators, all associations being positive and direct. In addition, statistically significant differences (*p* < 0.005) were obtained between Emotional Intelligence and Task Climate (*r* = 0.39), while differences with Ego Climate were not found. Statistically significant differences were observed between global levels of Emotional Intelligence and both anxiety dimensions, being negative and indirect as much for Trait-anxiety (*r* = −0.18; *p* = 0.003) as for State-anxiety (*r* = −0.13; *p* = 0.042).

## 4. Discussion

This research was carried out using a multigroup structural equation analysis in order to contrast associations between motivational climate in sport, emotional intelligence, and levels of anxiety depending on the type of sports practised. The route model developed achieves good fit indices, forming an acceptable explanatory model which enables the understanding of relations between motivational factors, emotional factors and anxiety in Spanish athletes, such as diverse studies in the national and international contexts have carried out [35,36,37,38,39,40].

Analysing motivational climate, the proposed structural model reveals a significant and inverse relationship between task climate and ego climate in individual and team sports, being more differentiated in team sports. It is evident that athletes who perceive a strong implication towards task climate encouraged by coaches, also perceive a low implication towards ego climate [41,42,43,44]. This is due to the fact that coaches train their athletes through a predominant orientation, either task orientation, rewarding effort and personal self-improvement, or ego orientation, encouraging rivalry between members of the same group and sheer demonstration of abilities [45]. In team sports, this negative relationship shows greater strength because athletes in individual sports get higher values in ego orientation and lower scores in task orientation than athletes. This can be explained by the greater group cohesion in team sports which encourage cooperation between members of the group, while outperforming others and demonstrating abilities that are more highly valued in individual sports [46,47,48].

Regarding category configuration of task climate dimensions, the category important role is the most influential for individual sports, while in team sports it is effort/improvement that correlates more strongly. In the same way, the influence of indicators of ego climate follows a similar proportion in individual and team sports, being rivalry between members of the group the most influential indicator, although it is higher in individual sports. This data finds its proof in contributions by Lenor et al. [49] and Tyler & Cobbs [50], as they indicate that rivalry between members of the group exists in any team sport, trying to stand out among others, whereas in individual sports, this factor gains greater importance because athletes compete alone against rivals.

In a similar way, influence of indicators in global levels of emotional intelligence follows the same tendency in individual and team sports. Nevertheless, in the latter the strength of correlation is slightly higher in all variables and especially in the dimension emotion utilisation. In this case, it is shown how athletes in collective sports are better at managing and using emotions than the ones who practise individual sports [51]. These results can be explained regarding higher socialisation between members of a team in collective sports, where greater interaction between them is needed, the athlete therefore acquires better abilities with regard to management and utilisation of emotions [52,53,54,55].

With regard to levels of anxiety according to motivational climate, a positive and direct relationship was found between task climate and trait-anxiety in athletes of individual sports, whereas in team sports this relationship is not significant. In the case of state-anxiety, the connection is indirect, showing greater strength in collective sports. Concerning ego climate, it is positively related with state-anxiety in individual sports. These data show that when the athlete adopts a predominant orientation towards task, their levels of state-anxiety fall, whereas in individual sports levels of trait-anxiety rise [56]. These data can be understood because the athlete that focuses on effort and personal self-improvement is also going to focus on the process, giving little importance to result. Therefore, their anxiety levels will not be so high as competition or training will not be considered to be stressful [57,58]. However, there is a direct connection with trait-anxiety in individual sports, understanding that this is produced by the fact that athletes with more anxious personalities can adopt an orientation towards task as a strategy to confront competition, considered more stressful in individual sports than in team sports [59,60].

Levels of state-anxiety and trait-anxiety are positively and directly related in both types of sports, although it is more stressed in individual sports. This connection is due to the fact that individuals with an anxious personality will reveal higher levels of state-anxiety while performing everyday situations, whereas athletes with low trait-anxiety will maintain lower levels of anxiety while confronting situations which can be perceived as threatening [60,61,62].

Analysing the relationship between motivational climate and emotional intelligence, it is proven that there is a direct connection with task climate, which is higher in individual sports. Likewise, the ego climate is positively related with emotional intelligence in individual sports. It becomes clear that athletes with better emotional abilities are able to orient themselves towards tasks, focusing on achieving internal rewards, thereby increasing extrinsic motivation [63,64,65]. This is due to the fact that athletes with higher emotional intelligence are able to focus on the process, giving priority to effort and personal self-improvement, which are more realistic and satisfactory goals than extrinsic ones [65,66].

Regarding the existing connection between emotional intelligence and anxiety, a significant, negative and indirect relation is found between state-anxiety and emotional intelligence in collective sports. Concerning the connection with trait-anxiety, it is negative in both categories of sports, showing greater strength in individual sports. These data can be explained by Ros et al. [67] in that instructions that emphasize the idea that emotional intelligence is an ability which can be learnt with the aim of controlling fundamental emotions in the sports context, such as stress, aggressiveness or anxiety, the latter being a disturbing element that deteriorates sport performance [52,68]. In collective sports, emotional intelligence rises and the levels of trait-anxiety fall; this is explained by the fact that in this category of sports, pressure endured by athletes during training sessions and competitions is distributed between members of the team [69]. In addition, levels of state-anxiety are negatively related with emotional intelligence in both categories, due to the fact that in sports individuals confront stressful situations that will raise their levels of anxiety, and they are able to control this situation if they improve their emotional abilities [70,71,72].

This research has a number of limitations, amongst which it is worth highlighting the scarce representation of athletes in the individual with contact category, besides the nature of the study, which does not allow for the establishment of cause-effect relationships, as it is a cross-sectional survey. Due to these reasons and regarding the data provided, in the future this research aims to setup intervention programs oriented towards reducing levels of anxiety through emotional intelligence and task-orientations in motivational climates, as well as registering data in different periods throughout the sport season, in order to contrast data in training sessions, precompetitive periods and competitions.

## 5. Implications for Practice

Based on the results obtained from the relationships given between motivational climate, emotional intelligence and anxiety, it seems interesting to develop some strategies for the improvement of these psychological factors and the prevention of anxiety in athletes regardless of the sport practiced.

The first point is the importance of promoting task-oriented motivational climates through actions that reward effort, group work and more self-determined motivations, being the role of psychologists and trainers extremely important. For this aim, it is relevant to use appropriate communication tools such as a positive feedback, promoting high but achievable goals, employing inquiry, rewarding effort, grouping athletes with others of similar levels, to establish shaping considering the implication of athletes or use of extrinsic reinforcements in moderation. Another strategy is the Task, Authority, Recognition, Group, Evaluation, Time (TARGET) method, which is an intervention system that coaches can use to generate task-oriented motivational climates, designing activities that involve personal challenges, conceiving errors as part of learning and allowing the athlete to make decisions recognizing the effort.

With regard to emotional intelligence, work can be done to reduce levels of anxiety using techniques from cognitive and behavioral psychology to perceive, understand and reorient emotions. In this line, positive affirmations must be created about situations that are interpreted as threatening. In addition, it is important to identify thoughts in order to reorient negative emotions. It is also important to practice mentally problematic situations and changes of perspective in order to approach problems on one’s own and to achieve personal growth. All this would help treat anxiety in a preventive way.

Finally, to work on anxiety in a specific way, an exhaustive follow-up must be carried out to control its levels. When these are relatively high, psychological tools based on programs that address relaxation patterns should be applied, such as the Jacobson technique, Schultz autogenic training, visualization techniques, positive self-talk or thought inversion.

## 6. Conclusions

There is an inverse relation between ego climate and task climate, which is stronger and more differentiated in collective sports, having an important role is the strongest indicator in task climate in individual sports, whereas in collective sports it is cooperative learning. Regarding ego climate, the strongest indicator is rivalry between members of the group. This indicator exerts greater influence in individual sports than in collective sports.

Emotional intelligence dimensions correlate more strongly in collective sports than in individual ones; a direct relation exists between task climate and emotional intelligence, which is higher in individual sports; ego climate relates positively and directly with emotional intelligence in individual sports, a negative and indirect relation between state and trait anxiety and emotional intelligence is found.

Trait-anxiety and task climate present a direct and positive relation in individual sports, whereas the connection with state-anxiety is negative and indirect in collective and individual sports; regarding relation between ego climate and state-anxiety, these variables hold a positive and direct relation in individual sports, and there exists a positive and direct connection between state-anxiety and trait-anxiety, which is more noticeable in individual sports.

## Figures and Tables

**Table 1:** Structural model in individual sports.

Relations between Variables			R.W.				S.R.W.
			Estimations	E.E.	C.R.	P	Estimations
SA	←	EC	2.77	0.75	3.65	***	0.16
SA	←	TC	−2.61	0.70	−3.71	***	−0.16
TA	←	TC	1.75	0.39	4.43	***	0.12
TA	←	SA	0.74	0.02	32.00	***	0.81
TA	←	EC	0.88	0.42	2.08	0.037 *	0.06
EI	←	SA	0.00	0.00	1.31	0.190	0.08
EI	←	TA	−0.01	0.00	−6.50	***	−0.42
EI	←	TC	0.29	0.02	10.90	***	0.47
EI	←	EC	0.07	0.02	2.69	0.007 **	0.11
E/I	←	TC	0.85	0.02	30.83	***	0.91
IR	←	TC	1.04	0.03	29.06	***	0.88
MR	←	EC	1.00	-	-	-	0.74
UR	←	EC	1.22	0.06	20.11	***	0.91
PM	←	EC	1.00	0.05	19.80	***	0.85
CL	←	TC	1.00	-	-	-	0.89
HEM	←	EI	0.93	0.03	26.58	***	0.85
SEM	←	EI	0.93	0.03	27.24	***	0.86
EP	←	EI	1.00	-	-	-	0.89
EU	←	EI	0.71	0.04	16.65	***	0.63
EC	↔	TC	−0.09	0.02	−3.90	***	−0.19

Note 1: TC, Task Climate; CL, Cooperative Learning ; E/I, Effort/Improvement; IR, Important Role; EC, Ego Climate; MR, Member Rivalry; PM, Punishment for Mistakes; UR, Unequal Recognition; SA, State Anxiety; TA, Trait Anxiety; EI, Emotional Intelligence; HEM, Heteroemotional Management; SEM, Self-emotional management; EP, Emotion Perception; EU, Emotion Utilisation. Note 2: R.W., Regression Weights; S.R.W., Standardised Regression Weights; E.E., Estimation Error; C.R., Critical Ratio. Note 3: *** Statistically significant relation between variables in level 0.005; ** Statistically significant relation between variables in level 0.01; * Statistically significant relation between variables in level 0.05.

**Table 2:** Structural model in team sports.

Relations between Variables			R.W				S.R.W
			Estimations	E.E.	C.R.	P	Estimations
SA	←	EC	2.90	0.81	3.58	***	0.16
SA	←	TC	−4.15	0.60	−6.89	***	−0.30
TA	←	TC	−0.29	0.42	−0.69	0.489	−0.02
TA	←	SA	0.80	0.02	27.49	***	0.79
TA	←	EC	−0.72	0.54	−1.33	0.182	−0.04
EI	←	SA	−0.00	0.00	−2.03	0.042 *	−0.13
EI	←	TA	−0.00	0.00	−2.96	0.003 **	−0.18
EI	←	TC	0.27	0.03	8.97	***	0.39
EI	←	EC	0.06	0.03	1.59	0.111	0.07
E/I	←	TC	0.89	0.02	34.22	***	0.90
IR	←	TC	1.00	0.03	33.07	***	0.89
MR	←	EC	1.00	-	-	-	0.58
UR	←	EC	1.51	0.10	14.05	***	0.92
PM	←	EC	1.35	0.09	14.28	***	0.87
CL	←	TC	1.00	-	-	-	0.93
HEM	←	EI	0.93	0.03	30.69	***	0.87
SEM	←	EI	0.88	0.02	32.81	***	0.90
EP	←	EI	1.00	-	-	-	0.92
EU	←	EI	0.87	0.04	21.16	***	0.72
EC	↔	TC	−0.09	0.01	−5.02	***	−0.25

Note 1: TC, Task Climate; CL, Cooperative Learning ; E/I, Effort/Improvement; IR, Important Role; EC, Ego Climate; MR, Member Rivalry; PM, Punishment for Mistakes; UR, Unequal Recognition; SA, State Anxiety; TA, Trait Anxiety; EI, Emotional Intelligence; HEM, Heteroemotional Management; SEM, Self-emotional management; EP, Emotion Perception; EU, Emotion Utilisation. Note 2: R.W., Regression Weights; S.R.W., Standardised Regression Weights; E.E., Estimation Error; C.R., Critical Ratio. Note 3: *** Statistically significant relation between variables in level 0.005; ** Statistically significant relation between variables in level 0.01; * Statistically significant relation between variables in level 0.05.
